# Dual-Reporter Mycobacteriophages (Φ^2^DRMs) Reveal Preexisting *Mycobacterium tuberculosis* Persistent Cells in Human Sputum

**DOI:** 10.1128/mBio.01023-16

**Published:** 2016-10-25

**Authors:** Paras Jain, Brian C. Weinrick, Eric J. Kalivoda, Hui Yang, Vanisha Munsamy, Catherine Vilcheze, Torin R. Weisbrod, Michelle H. Larsen, Max R. O’Donnell, Alexander Pym, William R. Jacobs

**Affiliations:** a Department of Microbiology and Immunology, Albert Einstein College of Medicine, New York,New York, USA; b Howard Hughes Medical Institute, Albert Einstein College of Medicine, New York, New York, USA; c KwaZulu-Natal Research Institute for TB/HIV (K-RITH), Durban, South Africa; d Department of Medicine, Albert Einstein College of Medicine, New York, New York, USA; e Department of Genetics, Albert Einstein College of Medicine, New York, New York, USA; f Division of Pulmonary, Columbia University Medical Center, New York, New York, USA; g Department of Epidemiology Allergy and Critical Care Medicine, Columbia University Medical Center, New York, New York, USA; h Centre for AIDS Programme of Research in South Africa (CAPRISA), Durban, South Africa; i Africa Centre for Health and Population Studies, University of KwaZuluNatal, Mtubatuba, South Africa

## Abstract

Persisters are the minor subpopulation of bacterial cells that lack alleles conferring resistance to a specific bactericidal antibiotic but can survive otherwise lethal concentrations of that antibiotic. In infections with *Mycobacterium tuberculosis*, such persisters underlie the need for long-term antibiotic therapy and contribute to treatment failure in tuberculosis cases. Here, we demonstrate the value of dual-reporter mycobacteriophages (Φ^2^DRMs) for characterizing *M. tuberculosis* persisters. The addition of isoniazid (INH) to exponentially growing *M. tuberculosis* cells consistently resulted in a 2- to 3-log decrease in CFU within 4 days, and the remaining ≤1% of cells, which survived despite being INH sensitive, were INH-tolerant persisters with a distinct transcriptional profile. We fused the promoters of several genes upregulated in persisters to the red fluorescent protein tdTomato gene in Φ^2^GFP10, a mycobacteriophage constitutively expressing green fluorescent protein (GFP), thus generating Φ^2^DRMs. A population enriched in INH persisters exhibited strong red fluorescence, by microscopy and flow cytometry, using a Φ^2^DRM with tdTomato controlled from the *dnaK* promoter. Interestingly, we demonstrated that, prior to INH exposure, a population primed for persistence existed in *M. tuberculosis* cells from both cultures and human sputa and that this population was highly enriched following INH exposure. We conclude that Φ^2^DRMs provide a new tool to identify and quantitate *M. tuberculosis* persister cells.

## IMPORTANCE

Tuberculosis (TB) is again the leading cause of death from a single infectious disease, having surpassed HIV. The recalcitrance of the TB pandemic is largely due to the ability of the pathogen *Mycobacterium tuberculosis* to enter a persistent state in which it is less susceptible to antibiotics and immune effectors, necessitating lengthy treatment. It has been difficult to study persister cells, as we have lacked tools to isolate these rare cells. In this article, we describe the development of dual-reporter mycobacteriophages that encode a green fluorescent marker of viability and in which the promoters of genes we have identified as induced in the persister state are fused to a gene encoding a red fluorescent protein. We show that these tools can identify heterogeneity in a cell population that correlates with propensity to survive antibiotic treatment and that the proportions of these subpopulations change in *M. tuberculosis* cells within human sputum during the course of treatment.

## INTRODUCTION

Heterogeneity in the response of bacterial populations to antibiotic treatment was observed soon after the initial use of penicillin, the first antibiotic discovered, to treat deadly staphylococcal and other infectious diseases (1, 2). Bigger reported that, while penicillin lysed most cells of a staphylococcal culture, a small fraction of cells, termed “persisters,” survived and although not heritably drug resistant, were phenotypically drug tolerant in that when regrown and retreated, the culture yielded survivors at a frequency similar to that of the parental population (3). McCune and Tompsett first described the phenomenon of persistence in *Mycobacterium tuberculosis* in 1956 (4). They observed a significant reduction in tubercle bacilli in the lung and spleen of mice in the first few weeks of monotherapy or treatment with any combinatorial pair of antibiotics, following which the census remained constant. The only exceptions were the use of pyrazinamide (PZA) with either isoniazid (INH) or streptomycin; these combinations cleared ~1 × 10^6^ to 3 × 10^6^ tubercle bacilli from both spleen and lung after 12 weeks of chemotherapy (4, 5). However, 3 to 4 months after the end of treatment, tubercle bacilli with drug susceptibility patterns identical to that of the parent strain were detected again in spleen or lung, highlighting the difficulty in eradicating mycobacterial infections (5, 6). These results, in part, indicated that *M. tuberculosis* has evolved mechanisms to avoid killing by antibiotics and immune effectors by entering into distinct physiological states (persistence) and host niches and that this surviving population is a major barrier to the timely cure of tuberculosis (TB) patients.

Standard short-course chemotherapy of human TB requires a combination of four different antibiotics for at least 6 months (7). In the first phase of treatment, *M. tuberculosis* is rapidly cleared, and most patients improve within a few weeks of treatment initiation. During the second phase, as in the mouse model, bactericidal activity is dramatically reduced, and lengthy treatment is required to eliminate persisters (8). *M. tuberculosis* persistence is an important contributing factor to the continuing global pandemic of TB (9), necessitating prolonged treatment that often results in more frequent treatment interruptions, treatment failures, and the development of drug resistance. The emergence of multidrug-resistant (MDR) TB and extensively drug-resistant (XDR) TB strains is due in large part to persistence. Despite the role of persistence in exacerbating the difficulty of TB control efforts, the molecular basis of *M. tuberculosis* persistence in humans remains poorly understood, the major limitation being the absence of a quantifiable *ex vivo* model of *M. tuberculosis* persistence. Our previous work suggests that *M. tuberculosis* persisters are a heterogeneous population with members that respond differently to stressful conditions to achieve a persistent or drug-tolerant phenotype (10–12). This study highlights the abundance of heterogeneity in laboratory and clinical *M. tuberculosis s*amples and describes a novel way to identify cells that are more likely to persist*.*

## RESULTS

### Transcriptional profiling of *M. tuberculosis* persisters.

**FIG 1:**
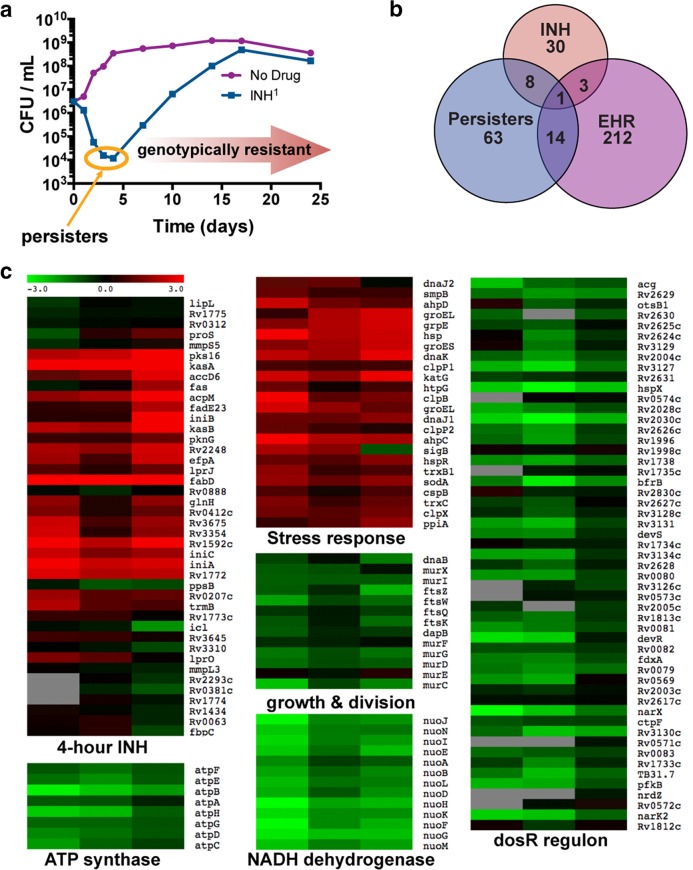
INH-sensitive survivors of INH treatment induce a transcriptional program consistent with a slow-growing stress-resistant state. (a) Treatment of *M. tuberculosis* with INH kills >99% cells in 4 days, leaving a largely INH-sensitive population from which an INH-resistant population emerges. (b) Survivors of 4 days of INH treatment induce genes consistent with INH sensitivity; the persister stimulon includes a subset of the enduring hypoxic response (EHR). Persister stimulon is defined here narrowly as genes statistically significantly induced >twofold at a false discovery rate (FDR) of <0.4% by SAM. EHR and INH stimulons were similarly defined by Rustad et al. (21) and Fu (18). (c) Heat maps of the persister stimulon in biological triplicates mapped onto genes induced by 4-h INH treatment, stress-responsive genes, genes involved in growth and cell division, the ATP synthase, the type I NADH dehydrogenase, and the *dosR* regulon. The scale indicates ±3-log_2_-fold changes relative to expression at day 0.

We developed a drug treatment model in which *M. tuberculosis* persisters are enriched by treating logarithmic-phase cultures with the lytic bactericidal antibiotic INH (3, 13). CFU analysis of *M. tuberculosis* cultures treated with 1 µg/ml INH, which is 20 times the MIC, indicated that 99.0 to 99.9% of the initial inoculum was nonviable at the end of 4 days (Fig. 1a). The surviving population (≤1%) was INH sensitive and showed the same killing kinetics after regrowth followed by INH treatment (1 µg/ml). A drug-resistant population emerged from this surviving *M. tuberculosis* population after prolonged incubation of cells in INH-containing medium (Fig. 1a). Based on these results, the day 4 time point was considered optimal for enriching the *M. tuberculosis* population sensitive to INH while maintaining a drug-tolerant phenotype. Genes exhibiting significant differences in expression between this surviving drug-tolerant population and untreated cells were determined by microarray hybridization (Fig. 1b and c) and were categorized into two sets. The first set consists of genes for which the changes in relative expression at day 4 were similar to those of cells exposed to INH for only 4 h (Fig. 1b and c), as reported in several studies (14–18). Upregulation of genes such as the *iniBAC* operon, which encodes an efflux pump and confers drug tolerance by expelling INH and lowering intracellular antibiotic concentrations, highlights the importance of this first set in *M. tuberculosis* survival (19). Importantly, the induction of this first set indicates that the population is largely genotypically drug sensitive, as resistant mutants do not exhibit the gene expression pattern typical of sensitive strains exposed to INH (20).

The second set of genes showed significant expression changes after 4 days but not 4 h of INH treatment. Differential expression of these genes, marked as persisters in Fig. 1b, may enable cell survival during INH treatment, or these genes may simply be induced in the persister population without functional consequences for cell survival. The transcripts specifically modulated in the persister-enriched population reflect cells primed for stress. Genes involved in cell division and energy metabolism, including *ftsZ*, *atp*, and the *nuo* NADH dehydrogenase operon, are downregulated, suggesting a nondividing or slowly growing population. Interestingly, *dosR* and most of its regulon are also downregulated, in concordance with its expression in the enduring hypoxic response (EHR) (21) but in contrast to its upregulation in some hypoxic models of nonreplicating persistence (NRP) (22). Antioxidant, heat shock, and protease genes, such as *ahp*, *katG*, *sodA*, *hsp*, *dnaK*, *grpE*, *groE*, *clpB*, and *clpX*, were upregulated in persister cells, indicating an adaptive response that may help cells survive drug treatment. Overall, the persister stimulon shows some overlap with several other stress conditions and models of drug tolerance, suggesting that there are several ways to generate a persister cell, although the current study has the special attribute of empirically illuminating the transcriptional profile of *M. tuberculosis* persister cells.

### Probing *M. tuberculosis* by dual-reporter mycobacteriophages (Φ^2^DRMs).

Promoter fusion to biochemical reporters or fluorescent protein reporter genes, including green fluorescent protein (GFP), red fluorescent protein (RFP), and tdTomato (23), has been used extensively to understand promoter regulation in mycobacteria (24–26). We took a reverse genetic approach to identify and differentiate various persister subpopulations in *M. tuberculosis* by fusing the tdTomato coding sequence to the promoters of specific genes, delivering these reporter cassettes into *M. tuberculosis* cells by a TM4-based, temperature-sensitive mycobacteriophage (27), and using relative fluorescence to measure promoter activity. Using promoters induced in the persister state marks these cells fluorescently and allows them to be distinguished from the bulk population on the basis of relative tdTomato fluorescence. Persister enrichment by INH was used in this study, and therefore, the fluorescently marked cells are referred to as “likely persisters.” The persister phenotype can be confirmed by assessing the survival advantage of likely persisters in comparison to the survival of the remaining bulk population under various lethal conditions.

**FIG 2:**
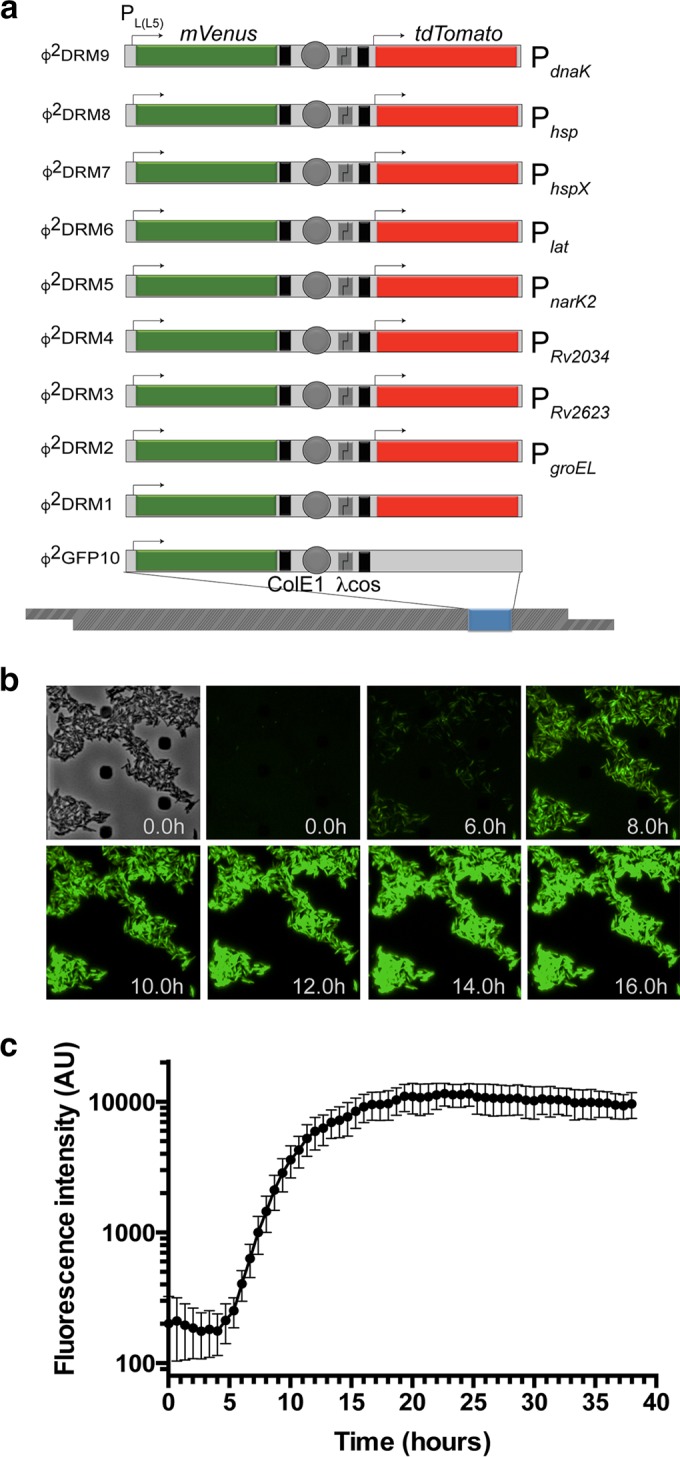
Design and fluorescence analysis of Φ^2^DRM constructs. (a) Schematics of the various Φ^2^DRMs used in the study. The expression of mVenus in Φ^2^GFP10 and all Φ^2^DRMs is driven from the constitutive P_L(L5)_ promoter. A unique promoter, listed on the right of each schematic, drives the expression of tdTomato in each Φ^2^DRM. Transcription terminators, placed upstream from tdTomato and downstream from mVenus to avoid transcriptional read-through, are indicated by black boxes. Arrows indicate the relative positions of promoters. (b) Expression kinetics of mVenus from P_L(L5)_ in *M. tuberculosis* by time lapse microscopy. Expression of mVenus was detectable after approximately 4 h of phage infection. (c) Quantitative plot of the kinetics of mVenus expression over time. Fluorescence intensity increased progressively over time, plateauing approximately 18 h after the addition of phage. AU, arbitrary units. The error bars represent SD of fluorescent intensity measured at three independent fields.

In order to construct Φ^2^DRMs, Φ^2^GFP10, a genetically engineered, temperature-sensitive TM4 mycobacteriophage that expresses the mVenus GFP from the P_Left_ promoter of mycobacteriophage L5 [P_L(L5)_] (27), was further modified by introducing a promoterless tdTomato gene into the reporter cassette to generate Φ^2^DRM1 (Fig. 2a). We selected promoters of genes whose expression was upregulated in and/or unique to several drug-tolerance models associated with enrichment of persister cells, including the 4-day INH treatment, hypoxic NRP (22), and nutrient limitation (28), and cloned these promoters upstream from the tdTomato gene in Φ^2^DRM1 (Fig. 2a).

The minimum time needed to detect fluorescence after transducing *M. tuberculosis* with the Φ^2^DRMs was determined by time lapse microscopy. The expression of mVenus from the reporter cassette common to both Φ^2^GFP10 and Φ^2^DRMs was observed starting 4 h after the initiation of transduction (Fig. 2b and c; see also Movie S1 in the supplemental material), with the mVenus fluorescence signal increasing steadily before plateauing after 16 h (Fig. 2b and c; see also Movie S1). mVenus expression can therefore be used as an indicator for the successful delivery of the fluorescent reporter cassette into *M. tuberculosis* cells shortly after phage transduction.

### Heterogeneity of gene expression in logarithmic-phase cultures of *M. tuberculosis*.

**FIG 3:**
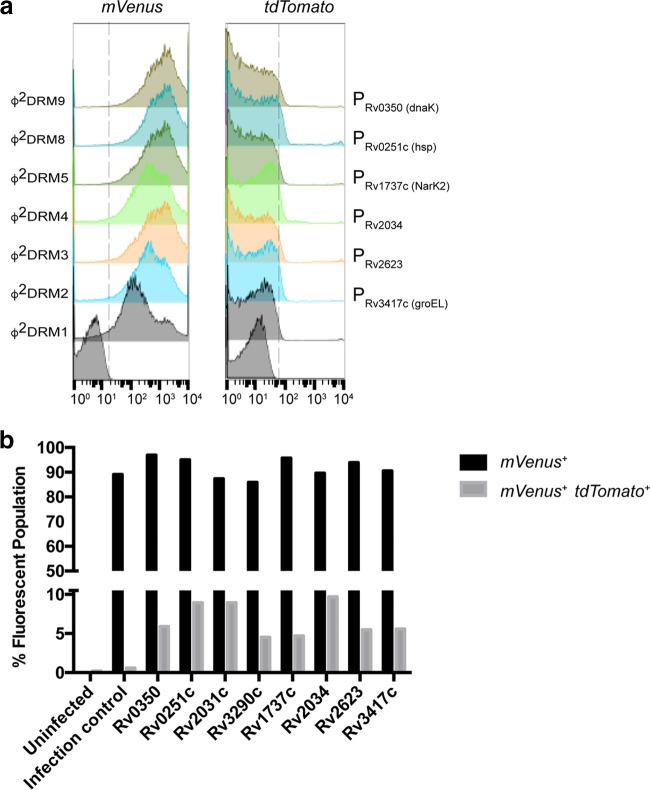
Fraction of *M. tuberculosis* cells expressing “persister upregulated genes” in logarithmic-phase cultures. (a) Left, log-phase *M. tuberculosis* cells infected with representative Φ^2^DRMs show similar distributions of mVenus expression; right, fractions of the parent populations expressing “persister upregulated genes” quantified by tdTomato expression. The promoter driving tdTomato expression is indicated on the right of each histogram. (b) Plot representing the percentages of mVenus^+^ and mVenus^+^ tdTomato^+^ populations after infection with individual Φ^2^DRMs. The double-positive cells indicate the fractions of cells expressing “persister upregulated genes” in logarithmic-phase *M. tuberculosis*. Promoters of the following genes were used to regulate tdTomato expression (the gene name or predicted function is indicated [http://tuberculist.epfl.ch/index.html]): *Rv0350*, *dnaK*; *Rv0251c*, *hsp*; *Rv2031c*, *hspX*, *acr*; *Rv3290c*, *lat*; *Rv1737c*, *narK2*; *Rv2034*, Ars repressor protein gene; *Rv2623*, universal stress protein family protein gene; *Rv3417c*, *groEL1*.

We next determined whether the genes upregulated in persisters are also differentially expressed in a portion of logarithmic-phase cells of *M. tuberculosis.* Following infection with each Φ^2^DRM, ~90% of the population expressed mVenus, and with comparable fluorescence intensities (Fig. 3a). These results indicated that (i) Φ^2^DRMs deliver recombinant DNA to at least 90% of the cell population capable of transcription and translation and (ii) the remaining ~10% of nonfluorescent cells were either not transduced by Φ^2^DRMs or had transiently or permanently lost the capacity for transcription/translation (Fig. 3a). The comparable levels of mVenus expression also suggested that most cells have similar transcription and translation potential.

Further analysis indicated that in the mVenus-positive (mVenus^+^) population, fewer than 10% of cells were also tdTomato positive (tdTomato^+^) (Fig. 3b). The variation in the percent tdTomato^+^ population indicated that there is a heterogeneity in the logarithmic-phase culture, i.e., cells experiencing the same microenvironment do not necessarily have consistent levels of expression of a given gene, and the differential expression of a specific gene between two cells within the same microenvironment possibly results from variation in the response to specific stimuli, stochastic fluctuations, or differences in their metabolic state.

**FIG 4:**
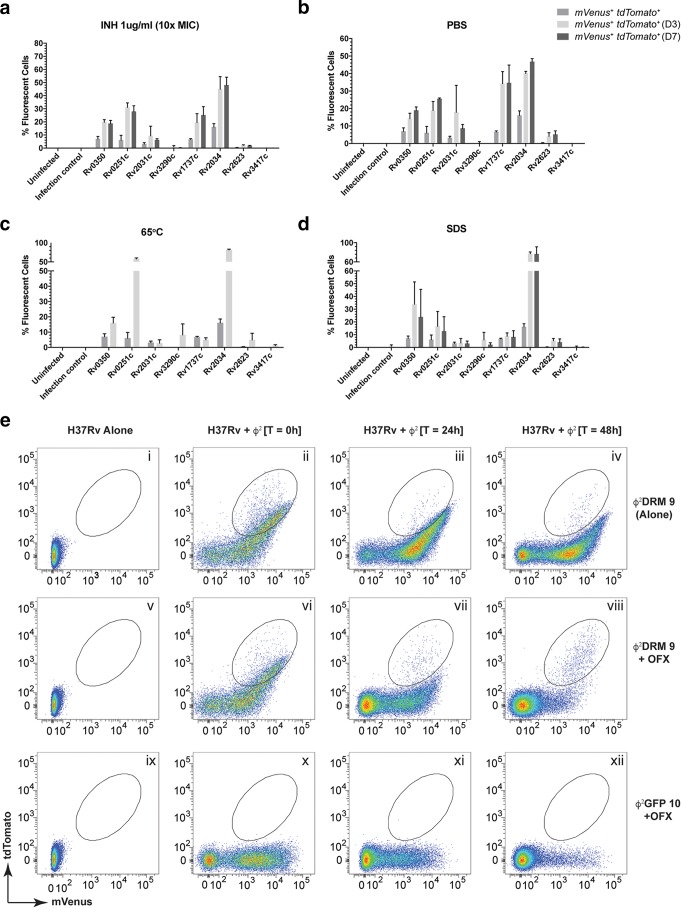
Activation of persister upregulated gene promoters in *M. tuberculosis* cells under stress. (a to d) The stress conditions used were INH (a), starvation (b), high temperature (c), and SDS (d). Gene promoters controlling tdTomato are indicated on each *x* axis. The percentage of the population turning on a specific promoter is plotted on each *y* axis. Error bars represent SEM from 3 independent experiments. For full gene names, see the legend to Fig. 3b. (e) Use of flow cytometry to monitor enrichment of a specific *M. tuberculosis* population after ofloxacin (OFX) treatment. Logarithmic-phase H37Rv was treated with OFX for 24 and 48 h and infected with either Φ^2^DRM9 (i to viii) or Φ^2^GFP10 (ix to xii). Cell-only controls (uninfected H37Rv) are shown in panels i, v, and ix. At time *t*_0_, a majority of the infected cells fluoresced green (ii, vi, and x). After 24 h of OFX treatment, the relative percentages of the fluorescent populations are reduced in the treated samples (compare panel vi to vii and x to xi). The same trend continued and the fluorescent populations were further reduced in the treated samples at 48 h (compare panel vi to viii and x to xii). At the end of 48 h, an mVenus^+^ tdTomato^+^ subpopulation (high tdTomato, low to medium mVenus) survives the treatment (circled, panel viii) and is the only fluorescent population remaining (compare panel iv to viii). The fluorescent population did not decrease in the untreated control cells (compare panels ii, iii, and iv).

To understand how stress conditions affect the expression of persister-specific stimulon genes, *M. tuberculosis* cells were infected individually with various Φ^2^DRMs and subjected to antibiotic treatment (INH), starvation (phosphate-buffered saline [PBS]), high temperature (65°C), or detergent (1% SDS), followed by analysis of mVenus^+^ and mVenus^+^ tdTomato^+^ populations at days 3 and 7 (Fig. 4). In untreated samples at time zero (*t*_0_), approximately ~7% of cells expressed *dnaK*/*Rv0350* and ~6% expressed *hsp/Rv0251c*. These *dnaK*/*Rv0350*^+^ and *hsp/Rv0251c*^+^ subpopulations increased to ~33.9% and ~16.6% after 3 days but then decreased to 23.8% and 12.6%, respectively, after 7 days of SDS exposure (Fig. 4d). Upon starvation, *M. tuberculosis* cells expressing *hspX/Rv2031*^+^ increased from ~3.1% to ~17.9% by day 3 and returned to ~8.6% by day 7 (Fig. 4b). Shifting to 65°C increased the percentages of cells expressing *dnaK*/*Rv0350* and *hsp*/*Rv0251c* to 15.9% and 57.6%, respectively (Fig. 4c), consistent with available transcriptomic data (28–31). These results indicate that when exposed to a stressful condition, all cells in a population do not respond by upregulating the same set of genes. Notably, Φ^2^DRMs provide us the ability to determine the fraction of a population that will respond to a particular stimulus (high temperature in this case) by expressing gene A versus gene B (e.g., 16% of cells expressing *dnaK*/*Rv0350* versus 57.6% expressing *hsp*/*Rv0251c*). Furthermore, the intensities of the individual responses and the mean expression of a particular gene can be estimated using fluorescence as a surrogate marker.

The above-described experiment provided strong evidence that there is heterogeneity not only in the logarithmic-phase culture but also in the cellular response to a given stress condition. The expression of a specific gene can occur transiently (if required for initial adaptation to attain a survival state) or throughout the stress exposure (if required to maintain the survival state). We hypothesized that since Φ^2^DRMs can deliver the reporter cassettes at the time needed, they can differentiate between these two conditions. To test this hypothesis, we used Φ^2^DRM9 as the test phage and ofloxacin (OFX) exposure as the stress condition. Logarithmic-phase strain H37Rv was treated with OFX (at 10× MIC) for 24 and 48 h before infection with Φ^2^DRM9 and analyzed by flow cytometry (Fig. 4e, panels i through viii). Φ^2^GFP10 was used as a control (Fig. 4e, panels ix through xii). At treatment initiation, most cells fluoresced green (Fig. 4e, panels ii, vi, and x), but by 24 h, the relative percentages of the fluorescent population dropped (Fig. 4e, compare panel ii to iii, vi to vii, and x to xi), and by 48 h, the majority of cells were nonfluorescent (Fig. 4e, compare panel ii to iv, vi to viii, and x to xii). By 48 h, a subpopulation expressing both high tdTomato and low to medium mVenus levels had survived the treatment (Fig. 4e, circled, panel viii) and was the only fluorescent population remaining (Fig. 4e, compare panel iv to viii). This subpopulation is also present in the untreated sample (Fig. 4e, circled, panels ii and vi) but is in a minority because of the presence of another double-positive population (high green and medium to high tdTomato), which is selectively eliminated by OFX treatment (Fig. 4e, compare panel iv to viii). A point to be noted is that Φ^2^DRMs are TM4-derived reporter phages, which have been used routinely to determine the susceptibility of *M. tuberculosis* against multiple drugs, including ofloxacin in various studies (27, 32, 33). Therefore, the elimination of signal was a result of the loss in cell viability and not because of the downregulation of Φ^2^DRM receptors on *M. tuberculosis* cells in the population that did not fluoresce. These results indicate that the cells enriched after OFX treatment express *dnaK* throughout the treatment period and that the Φ^2^DRMs are viable tools to identify the likely persister population.

### Functional implications of the differential expression of genes from the persister stimulon.

Time lapse microscopy in combination with microfluidics was used to determine whether the cells expressing tdTomato differed from the bulk population (see Movie S2 in the supplemental material). Based on the above-described results, we hypothesized that *M. tuberculosis* cells upregulating persister-induced genes would have a survival advantage under drug treatment, and so *M. tuberculosis* cells were transduced with Φ^2^DRM9, loaded into a microfluidic device, treated with 1 µg/ml INH, and imaged every 15 min for 3 days. Exposure of an *M. tuberculosis* cell to a bactericidal antibiotic would result in one of the following outcomes: (i) the cell would survive because it had a preexisting mutation or acquired one during antibiotic exposure, e.g., a mutation in the target or activator gene; (ii) the cell would survive because it was a preexisting persister before the initiation of INH treatment; (iii) the cell would survive because it elicited an adaptive response to antibiotic stress to become a persister and tolerate the antibiotic without acquiring a relevant genetic mutation; or (iv) the cell would die because it was neither a resistant mutant nor a persister, preexisting or induced.

**FIG 5:**
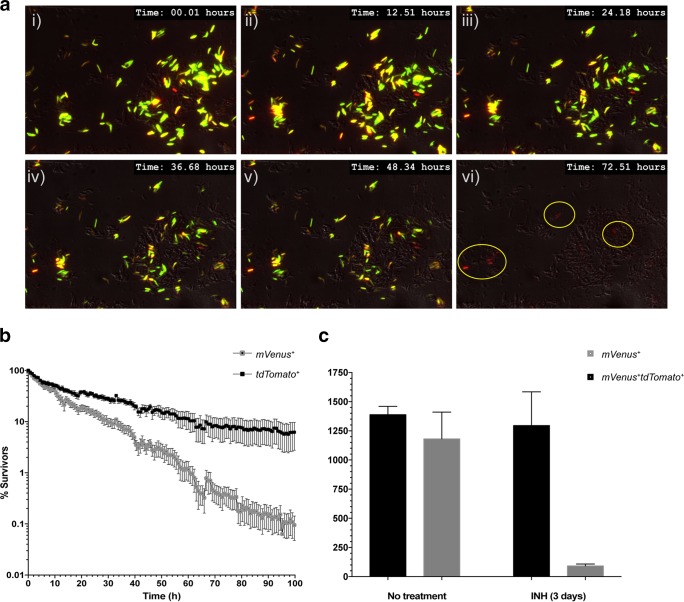
Identification of “likely persister” population in *M. tuberculosis* by Φ^2^DRMs. (a) Time lapse microscopy. Φ^2^DRM9-infected *M. tuberculosis* cells were treated with INH on a microfluidic device and imaged every 15 min for 3 days. Representative time frames from the time lapse movie at 12-h intervals are shown. At treatment initiation, most cells were mVenus^+^, with a few mVenus^+^ tdTomato^+^ cells. At the end of treatment, all surviving cells (circled) were tdTomato^+^ with very low mVenus fluorescence. (b) Survival kinetics of *M. tuberculosis* cells expressing high levels of *dnaK* promoter activity (black squares) in comparison to the bulk population (grey squares). The plot indicates that cells expressing *dnaK* were able to resist INH treatment longer than cells not expressing *dnaK*. Error bars represent SEM from 5 independent experiments. (c) Fifty thousand *M. tuberculosis* cells infected with Φ^2^DRM9 were sorted by flow cytometry for mVenus^+^ and mVenus^+^ tdTomato^+^ cells before and after INH treatment. The viable counts in the sorted samples were determined by CFU plating. *M. tuberculosis* cells expressing tdTomato survive preferentially after INH treatment in comparison to cells that express only mVenus. Error bars represent SEM from 3 independent experiments.

At the start of INH treatment, most of the cell population is only mVenus^+^, with a few double-positive (mVenus^+^ tdTomato^+^) cells. As the drug exposure continues, most cells start to lose viability, manifested as the loss of fluorescence (see Movie S2 in the supplemental material). Occasionally, the *dnaK* promoter was induced in a specific cell, turning it from tdTomato^−^ to tdTomato^+^. By 72 h, all surviving cells were double positive, with high tdTomato and low to medium mVenus expression (Fig. 5a; see also Movie S2). These results suggest that the persisters not only preexist but are induced by INH treatment. We next examined independent time lapse movies to evaluate differences in the death kinetics of the mVenus^+^ and mVenus^+^ tdTomato^+^ populations. The results indicated that the mVenus^+^ tdTomato^+^ subset withstood INH treatment longer than the overall mVenus^+^ population (Fig. 5b), confirming that cells with higher levels of *dnaK* promoter activity are more likely to tolerate INH treatment than cells expressing basal levels and thus represent the likely persister population.

We further examined why flow cytometric analysis of Φ^2^DRM9-infected *M. tuberculosis* cells detected only ~20% of the population expressing high levels of *dnaK* after 3 days of INH treatment, whereas time lapse microscopy indicated that almost the entire surviving population expressed high levels of *dnaK* under similar conditions. An equal number of mVenus^+^ and mVenus^+^ tdTomato^+^ *M. tuberculosis* cells infected with Φ^2^DRM9, before and after 3 days of INH treatment, were sorted by flow cytometry and the numbers of viable cells were determined by CFU plating. The results indicate that prior to INH treatment, relatively similar numbers of mVenus^+^ and mVenus^+^ tdTomato^+^ cells were viable (relative ratio, 0.85:1). However, after the INH treatment, the viability ratio of mVenus^+^ and mVenus^+^ tdTomato^+^ cells changed, and in comparison to the survival of mVenus^+^ cells, the mVenus^+^ tdTomato^+^ cells survived preferentially, with a relative ratio of 0.07:1, consistent with the time lapse microscopy results (Fig. 5c). This result indicates that a significant proportion of mVenus^+^ cells retain fluorescence after loosing viability when analyzed by flow cytometry, which is not the case in the time lapse-imaging experiment. This is likely because at the time of flow cytometry analysis, there is no photobleaching of mVenus in the nonviable *M. tuberculosis* cells. However, in a time lapse experiment, repeated exposures result in photobleaching, and if the protein is not replenished, the nonviable cells eventually lose fluorescence. This non-viable mVenus^+^ population can be eliminated if the cells are infected with Φ^2^DRM9 after INH treatment, as observed in Fig. 4e. Interestingly, in the time lapse-imaging experiment, a few of the surviving cells at the end of imaging have very low mVenus fluorescence, raising the possibility that in the persister cells, the P_L(L5)_ promoter is inactive even in the absence of L5 repressor and that this phage promoter has a more complex regulation than previously envisioned.

### Differential expression of genes in *M. tuberculosis* from human sputum samples.

**FIG 6:**
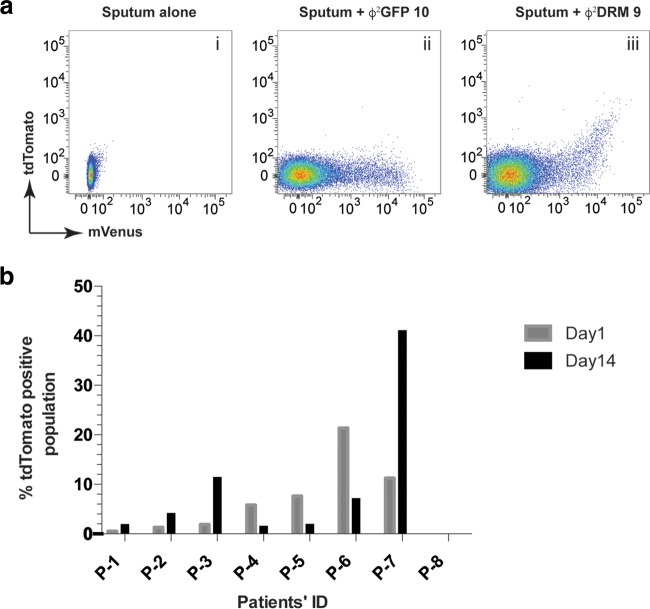
Identification of likely persister populations in sputum samples. (a) Sputum samples obtained from TB patients were infected with Φ^2^DRM9 and analyzed by flow cytometry. Phage Φ^2^GFP10 was used as an infection control. (b) Plot representing the fractions of mVenus^+^ tdTomato^+^ populations in sputum samples of TB patients at the time of disease presentation and after 2 weeks of TB treatment.

We next examined whether Φ^2^DRMs could identify changes in the fraction of *M. tuberculosis* cells expressing a persister-specific gene during the course of TB treatment. The bacillary loads in patient sputa were determined based on the number of fluorescence events; the mVenus fluorescence intensity distribution was comparable between Φ^2^GFP10- (27, 34) and Φ^2^DRM9-infected samples, confirming Φ^2^DRM’s ability to detect *M. tuberculosis* in clinical material (Fig. 6a, compare panels ii and iii). Additional sputum samples were collected from 20 newly diagnosed TB patients prior to the initiation of TB treatment (day 0) and after 2 weeks of treatment (day 14), and the samples were then infected with Φ^2^DRM9. The fractions of mVenus^+^ tdTomato^+^ cells relative to total mVenus^+^ cells at day 0 and day 14 are shown for representative samples (Fig. 6b). In 4 of 8 samples, the relative percentage of tdTomato+ cells increased by day 14, and in 3 out of 8 samples, the relative percentage of tdTomato+ cells decreased by day 14. No tdTomato^+^ cells were identified in sample P-8. These data led us to hypothesize that the transcriptional profile of *M. tuberculosis* in TB patients undergoing treatment is dynamic and therefore likely to influence treatment outcome.

## DISCUSSION

Phenotypic heterogeneity enables microorganisms to survive sudden environmental changes and to persist during adaptation periods without acquiring any genetic mutation. In general, the benefit phenotypic heterogeneity provides to an infectious agent impedes the effort to quickly treat infectious diseases, a particularly challenging problem with TB because phenotypic heterogeneity necessitates lengthier drug treatments of TB patients and may prevent the immune system from completely clearing *M. tuberculosis*, an added concern as only a few bacteria are sufficient to cause disease (35). In this study, we developed tools to identify *M. tuberculosis* cells that are more likely to persist. The identification and characterization of persisters could facilitate the development of means to shorten TB chemotherapy.

We generated various Φ^2^DRMs using the promoters of genes that are induced in *M. tuberculosis* cells surviving 4 days of INH treatment or in other models of *M. tuberculosis* persistence (22, 28). The variation in the proportions of mVenus^+^ tdTomato^+^ *M. tuberculosis* populations identified using the various Φ^2^DRMs suggests that the individual Φ^2^DRMs distinguished several, probably overlapping likely persister populations (Fig. 3 and 4). These data also indicate that the persister population enriched by day 4 of INH treatment is not a homogeneous population, consistent with the hypothesis that there are multiple ways to become a persister. Phenotypic heterogeneity is a complex trait selected during evolution to enhance the survival of clonal communities (36). For example, in *Escherichia coli*, the *cka*-encoded colicin K toxin provides a competitive advantage by lysing cells that do not express the immunity-encoding *cki* gene. *cka* is induced ~20-fold during stationary phase (37), but only in 3% of the population (38), whereas *cki* is expressed in a larger population to prevent self-lysis. Similarly, in *Myxococcus xanthus*, the developmental regulator gene *devR*, required for fruiting body formation, is expressed only in a subpopulation (39), and in *Bacillus subtilis* exposed to appropriate environmental conditions, the threshold level of active Spo0A dimer required to initiate sporulation is also achieved in only a small subpopulation (40, 41). These results indicate that the expression level of a protein can strongly influence phenotype. The bimodal distribution of a gene’s expression may affect single or multiple phenotypes. For example, in *M. tuberculosis*, the expression of *katG*, encoding the catalase peroxidase that is the activator of the prodrug INH, correlates inversely with INH sensitivity. Cells that express *katG* are peroxide tolerant and INH sensitive, whereas cells that do not express *katG* are transiently peroxide sensitive and INH tolerant (42). Therefore, the bimodal distribution of *katG* expression between cells in the same microenvironment results in phenotypic heterogeneity in a genotypically homogeneous population.

Importantly, the Φ^2^DRMs we developed can determine both the fraction of cells expressing a specific gene and the cell-to-cell variation in the expression of that gene within the fluorescence-positive subset. An advantage of Φ^2^DRMs is the flexibility to choose the time of reporter gene delivery and the gene promoter controlling the expression of the fluorescent protein. Even though all of the Φ^2^DRMs are nonpermissive for the production of viable phages at 37°C or above, it is possible that some of the persister-induced genes are also upregulated in response to the initial phage shock experienced by *M. tuberculosis* during Φ^2^DRM infection. *E. coli* encodes a specialized phage shock protein (PSP) operon to deal with phage-induced stress, and *psp* was initially discovered as an upregulated gene induced by the phage *gene IV* protein during filamentous phage f1 infection of *E. coli* (43). However, the *psp* operon is not only upregulated during f1 phage infection; it is also transiently induced by a variety of membrane-altering stresses, including extreme heat shock (50°C), hyperosmotic shock, and ethanol treatment (10% ethanol). Considering the fact that less than 10% of the population express persister-induced genes in the logarithmic phase 16 h after Φ^2^DRM infection, which does not increase significantly on further incubation of 24 to 48 h in the absence of any other external stress (data not shown), we do not expect a significant influence of Φ^2^DRM-induced phage shock response on the results presented here. RNA-seq analysis of *M. tuberculosis* after Φ^2^DRM infections, at temperatures that are permissive (30°C) and nonpermissive (37°C) for phage amplification, will provide further insights on the phage shock response in *M. tuberculosis.* Nevertheless, Φ^2^DRMs provide the flexibility to monitor the expression levels of multiple genes in a sample after incubation with different Φ^2^DRMs. One of the primary advantages of Φ^2^DRMs over episomal or integrative plasmids is their applicability to examining both laboratory and clinical *M. tuberculosis* samples. Φ^2^DRMs can be used directly on clinical samples from TB patients, providing the means to stratify *M. tuberculosis* cells in the clinical sample on the basis of expression profile prior to downstream analyses, such as transcriptomics and metabolomics. Recently, the transcriptomic profile of the bulk population of *M. tuberculosis* present in patient sputa has been reported (44). These data will be very useful in determining the vulnerabilities of the pathogen in the context of infection. And yet, given the observed heterogeneity among the infecting cells, our interest should be focused on the recalcitrant persister population. Considering that persisters are a minor subpopulation, the enrichment of *M. tuberculosis* samples for persisters will reveal the unique characteristics distinguishing persisters from the bulk population. The use of Φ^2^DRMs on serial sputum samples could allow the identification of clinically relevant biomarkers of TB treatment responses, and if validated, these biomarkers might also serve as surrogate indicators for clinical outcome. A combined use of Φ^2^DRMs, dielectrophoresis-based cell separation (45), and single-cell transcriptomics (46) would help identify biologically meaningful differences in these subpopulations, without the relevant characteristics being averaged out or diluted by the overwhelming number of nonpersister cells. Further work on Φ^2^DRMs, such as probing clinical specimens from TB patients with Φ^2^DRMs during the course of antitubercular treatment, in conjunction with transcriptomic analysis, will provide valuable insights into the biology of *M. tuberculosis*, potentially advancing the quest to shorten the duration of tuberculosis treatment.

## MATERIALS AND METHODS

### Model of persistence.

*M. tuberculosis* was grown to an optical density at 600 nm (OD_600_) of 0.2, diluted 1:10 into 5 ml 7H9 broth supplemented with 10% oleic acid-albumin-dextrose-catalase (OADC), 0.5% glycerol, and 0.05% Tween 80, and grown in inkwell bottles with shaking at 37°C. Cultures were treated with 1 µg/ml INH, and CFU counts were monitored by plating on 7H10 medium containing OADC and 0.5% glycerol at days 1, 2, 3, 4, 7, 10, 14, 16, and 24.

### Transcription profiling of *M. tuberculosis* persisters.

Triplicate 60-ml cultures of *M. tuberculosis* strain mc^2^7000 (47) in 7H9 medium containing 10% OADC, 0.5% glycerol, 50 µg/ml pantothenate, and 0.05% Tween 80 were grown to an OD_600_ of 0.2 in 500-ml roller bottles in a roller incubator at 37°C. Ten-milliliter aliquots were removed to 15-ml conical tubes and centrifuged for 10 min at 5,000 × *g*. The supernatant was poured off, and the cell pellet was resuspended in 1 ml Qiagen RNAprotect reagent and incubated for 4 h at room temperature before storage at −80°C. The remaining 50-ml cultures were treated with 1 µg/ml INH and returned to the incubator for 4 days. On the fourth day, the cultures were collected by centrifugation, resuspended in 2 ml of RNAprotect, and incubated for 4 h at room temperature before storage at −80°C. Cells were collected by centrifugation and resuspended in 1 ml buffer RLT from a Qiagen RNeasy kit. The suspension was transferred to FastPrep blue-cap tubes and processed for 45 s at a speed of 6 m/s in a FastPrep apparatus. After a brief incubation on ice, the debris was removed by centrifugation, and the supernatant (~750 µl) was removed to a fresh microcentrifuge tube. Five hundred microliters of absolute ethanol was added, and the samples were applied to RNeasy columns in two applications. At this point, the RNA was purified as recommended by the Qiagen protocol, and contaminating DNA was removed with the Ambion Turbo DNA-free kit, according to the manufacturer’s instructions. RNA yield, purity, and integrity were checked on a NanoDrop spectrophotometer and an Agilent Bioanalyzer 2100 microfluidic system. For microarray hybridization, cDNA probes were prepared by incubating 2 µg RNA with 2 µl Random Hexamers (3 mg/ml) and 1 µl RNaseOUT (Invitrogen) in an 18.5-μl reaction volume for 10 min at 70°C. Samples were then placed on ice for 1 min before a brief centrifugation on a tabletop centrifuge to collect condensation. The following components were added to the reactions: 6 µl 5× First Strand buffer, 3 µl 0.1M DTT, 0.6 µl 25mM dNTP/aminoallyl-dUTP labeling mix [prepared by the addition of 47.6 µl 100mM dATP, dCTP, and dGTP, 28.5 µl dTTP, and 19.1 µl 0.1M KPO4 to a vial of 5-(3-aminoallyl)-dUTP (Sigma catalog no. A0410)] and 2 µl Superscript III reverse transcriptase (Invitrogen). The reactions were incubated in a 42°C water bath for 16 hours. Reactions were stopped by adding 10 µl each of 0.5 M EDTA and 1 M NaOH and incubating for 15 min at 65°C followed by a brief centrifugation to collect condensation. Tris (25 µl 1M pH 7.0) was added to bring the reaction to neutrality. Reactions were cleaned up with the Qiagen MinElute kit following the manufacturer’s instructions, except substituting 5 mM KPO4 pH 8.0 80% EtOH for the wash buffer and 4 mM KPO4 pH 8.5 for the elution buffer, and eluting twice with 30 µl elution buffer with 1-min incubations prior to centrifugation. The cDNA yield was quantitated on a Nanodrop spectrophotometer before drying on a SpeedVac vacuum concentrator. Pellets were dissolved in 4.5 µl 0.1M Na_2_CO_3_ pH 9.3 and 4.5 µl Cy3 or Cy5 dye (mono-reactive dye pack, dissolved in 73 μl DMSO [Amersham]) were added to reference and test samples, respectively. Labeling reactions were incubated for 1 hour in the dark before being neutralized with 35 µl 100 mM NaOAc pH 5.2 and cleaned up with the MinElute kit following the manufacturer's instructions, eluting twice with 30 µl of elution buffer. Labeling efficiency was evaluated on a Nanodrop before corresponding probes were combined and dried on a SpeedVac. Probes were hybridized to 70-mer oligonucleotide DNA microarrays representing the complete *M. tuberculosis* genome (v4), with 4 in-slide replicates (JCVI PFGRC). The slides were prehybridized in 5× SSC, 0.1% SDS, and 1% BSA filtered through a 0.2-μm filter in a Coplin jar for 1 hour at 42°C before being washed 8× with 2 min of shaking in a staining dish in MilliQ water. Slides were washed in isopropanol and dried by centrifugation for 10 min at 1000 rpm. Lifterslip coverslips (ThermoFisher) were placed on the slides in Corning hybridization chambers, and the probes that had been dissolved in 50 µl hybridization buffer consisting of 40% formamide, 5× SSC, 0.1% SDS, and 0.6 µg/μl Salmon Sperm DNA were placed on the slide after a 10-min denaturation at 95°C. Chambers were sealed and incubated in a 42°C water bath for 16 hours. Slides were washed 2× for 5 min in low stringency wash (2× SSC, 0.1% SDS, 100 µM DTT at 55°C), 2× for 5 min in medium stringency wash (0.1× SSC, 0.1% SDS, 100 µM DTT), and 2× for 5 min in high stringency wash (0.1× SSC, 100 µM DTT). Slides were rinsed with MilliQ water followed by drying by centrifugation for 1 min at 1000 rpm. Slides were scanned on a GenePix 4000A scanner and images were processed with the TM4 software suite. TIGR Spotfinder was used to grid and quantitate spots, and TIGR MIDAS was used for Lowess normalization, standard deviation regularization, and in-slide replicate analysis. The results were analyzed in MeV with Significance Analysis of Microarrays (SAM) and hierarchical clustering algorithms. Heat maps were prepared in MeV.

### Generation of Φ^2^DRMs.

Vector pYUB1551 was derived from pYUB1229 by cloning the mVenus gene downstream from the P_Left_ promoter from mycobacteriophage L5 [P_L(L5)_] (27, 48). The promoterless tdTomato gene (23) was cloned between the ClaI and PacI sites of pYUB1551 to generate pYUB1555. *M. tuberculosis* promoters upregulated in various drug-tolerant populations were cloned between the ClaI and SpeI sites (the primers used for cloning the promoter regions are provided in Table S1 in the supplemental material). The resulting constructs were then cloned in phAE159 by *in vitro* packaging to generate phasmids, followed by electroporation into *Mycobacterium smegmatis* strain mc^2^155 (51) to obtain the respective Φ^2^DRMs using the standard protocol described previously (27, 49). Individual plaques were picked into 200 µl of MP buffer (50 mM Tris [pH 7.6], 150 mM NaCl, 10 mM MgCl2, and 2 mM CaCl_2_) and incubated for 30 min at room temperature before amplification. High-titer phage lysates were obtained by phage amplification using the protocol described previously (27, 49). The phage lysates were supplemented with pantothenic acid (final concentration, 100 µg/ml) when used to infect the pantothenate auxotroph *M. tuberculosis* mc^2^6230 (27).

### Kinetics of gene expression in *M. tuberculosis* after mycobacteriophage infection.

The CellASIC ONIX microfluidic device (Millipore, Inc.) was used for all time lapse movies. *M. tuberculosis* cultures were grown to logarithmic phase (OD_600_ of 0.8 to 1.0) in 7H9 medium with 0.05% Tween 80 at 37°C. Cells were centrifuged (5,000 × *g* for 10 min at room temperature), washed twice with MP buffer, and resuspended in 7H9 medium, with no detergent, to an OD_600_ of 1.0. These cells were loaded at 10 lb/in^2^ for 20 s onto a CellASIC ONIX microfluidic plate (catalog no. B04A-03-5PK) that was preheated at 37°C. The cells trapped in the device were infected with Φ^2^GFP10 by injecting the phage [10^9^ PFU/ml] into the microfluidic device at 3 lb/in^2^ for 2 h at 37°C. Infection with Φ^2^GFP10 was terminated by switching the injection to 7H9 medium containing 0.05% Tween 80. The use of detergent inhibited superinfection during the period of observation in the time lapse movie. The expression kinetics of fluorescent protein was followed by capturing bright-field and fluorescent images every 15 min on a Nikon Eclipse Ti microscope. The fluorescent intensity was quantitated for the fluorescent images taken at different time points by creating three identical regions of interest (ROIs). Special attention was given to ensure that ROIs covered the same set of cells over time. Fluorescence intensity was estimated using the built-in feature in NIS-Element software (Advanced Research [AR] version 4.40.00).

### Identification of unique *M. tuberculosis* subpopulations using Φ^2^DRMs. (i) Detection in *M. tuberculosis* cultures using flow cytometry.

*M. tuberculosis* cells were prepared for infection with Φ^2^DRMs using the protocol described above. All the infections and incubations were carried out at 37°C unless otherwise indicated. Amounts of 50 µl of cells were incubated with individual Φ^2^DRMs at a multiplicity of infection (MOI) of 100:1, as described previously (27). After 16 h of infection with Φ^2^DRMs, the infection was terminated by the addition of an equal volume of 7H9 medium with 0.1% Tween 80; this is considered time zero. Next, all of the samples were divided into 5 equal parts. Samples were treated with INH (final concentration, 1 µg/ml) or SDS (final concentration, 1%) or were shifted to 65°C. For starvation, the Φ^2^DRM-infected *M. tuberculosis* cells were washed twice with PBS with 0.05% Tween 80 and incubated at 37°C. The samples were acquired by flow cytometry on the FACSAria II flow cytometer (BD Biosciences) at days 0, 3, and 7 and then analyzed using the FlowJo software package (version 10.0.7; TreeStar, Inc., Ashland, OR). *M. tuberculosis* cells expressing only one fluorescent protein, i.e., mVenus or tdTomato, were used as compensation controls. The Φ^2^DRM9-infected *M. tuberculosis* cells were sorted at time zero and at day 3 after INH treatment to collect 50,000 events, gating for mVenus^+^ and mVenus^+^ tdTomato^+^ cells on FACSAria II using the same gates as used for data acquisition as described above. Different dilutions were plated on 7H10 plates supplemented with 0.5% glycerol, 10% OADC, and 100 µg/ml pantothenic acid. The plates were incubated at 37°C for 4 weeks before counting CFU. The results from three independent experiments are plotted in Fig. 4a to c and 5c. For OFX treatment, the *M. tuberculosis* H37Rv cells were prepared as described above and resuspended in 7H9 medium, with no detergent, to an OD_600_ of 1.0. These cells were treated with OFX at 10× MIC (10 µg/ml) in a 100-µl reaction mixture at 37°C. Samples were taken out at time zero, 24 h, and 48 h, incubated with Φ^2^DRM9 at an MOI of 100, and further incubated for 12 h at 37°C. At the end of incubation, the samples were immediately acquired on the FACSAria II flow cytometer (BD Biosciences, CA), using identical settings. One hundred thousand events were collected and analyzed using the FlowJo software package (version 10.0.7; TreeStar, Inc.).

### (ii) Detection of likely persisters in sputum samples of TB patients.

The Prince Cyril Zulu Communicable Disease Clinic and King DinuZulu Hospital in Durban, South Africa, were used as clinical sites for the collection of sputum samples. A registered nurse collected the sputum samples from 20 newly diagnosed TB patients prior to the initiation of TB therapy and at 2 weeks into the treatment. The samples were processed using the standard *N*-acetyl-l-cysteine-NaOH (NALC) method (50). The samples were divided into two parts and were infected with Φ^2^GFP10 and Φ^2^DRM9 as described previously (27, 34). After 12 h of infection, the fluorescence data were acquired on the FACSAria II instrument (BD Biosciences) after compensation with *M. tuberculosis* expressing either mVenus or tdTomato. The data were analyzed using the FlowJo software package.

### (iii) Detection in *M. tuberculosis* culture using microfluidics.

*M. tuberculosis* cells were prepared for phage infection and loaded onto the CellASIC ONIX microfluidic device (Millipore, Inc.) as described above, except that the cells were infected with Φ^2^DRM9 for 12 h prior to loading onto the microfluidic device. The sample was treated with INH (1 µg/ml), and the changes in the expression of mVenus and tdTomato were recorded by capturing both bright-field and fluorescent images at 30-min intervals by time lapse microscopy. *M. tuberculosis* cells losing fluorescence after INH treatment were scored as nonviable, whereas cells maintaining fluorescence under identical conditions were scored as viable.

The relative percentages of mVenus^+^ and mVenus^+^ tdTomato^+^ populations in the time lapse movie were determined using the object count and “automated measurement results” functions in the NIS-Element software (AR version 4.40.00). The thresholding for both the mVenus and tdTomato intensity was performed by applying fixed threshold values at the beginning of INH treatment. These settings were kept constant across various experiments and were applied to all the images of a time lapse movie. The object count and the binary area for the mVenus and tdTomato channels were individually estimated at each time point after running the automated measurement results option. The percentages of mVenus- or tdTomato-positive populations at time *n* (*t_n_*) were calculated for >1,000 cells by estimating the percentage of the binary fluorescent area at *t_n_* over the binary fluorescent area at *t*_0_. The relative binary area was used to calculate the changes in the mVenus^+^ and tdTomato^+^ populations to avoid errors in estimating cell number because of the inability of the “object count” feature to differentiate cells touching each other. The mean values and standard errors of the means (SEM) for mVenus^+^ and tdTomato^+^ populations at each time point were calculated from multiple fields in at least five independent experiments.

### Accession number(s).

 Microarray data have been deposited in NCBI GEO under accession number GSE86141.

## SUPPLEMENTAL MATERIAL

Table S1  Primers used to construct Φ^2^DRMs. Table S1, DOCX file, 0.1 MB

Movie S1  Kinetics of mVenus expression after reporter mycobacteriophage infection. Download Movie S1, AVI file, 13.8 MB

Movie S2  Time lapse movie to show the enrichment of the INH-tolerant *M. tuberculosis* population using Φ^2^DRM9. Download Movie S2, AVI file, 2.5 MB
