# Supplementary material for: Dual-Reporter Mycobacteriophages (Φ2DRMs) Reveal Preexisting Mycobacterium tuberculosis Persistent Cells in Human Sputum
Source: mBio. 2016 Oct 25;7(5):e01023-16. doi: 10.1128/mBio.01023-16 (PMC5080378; doi:10.1128/mBio.01023-16)
Supplement: Table S1 — Primers used to construct Φ2DRMs. [file mbo005163031st1.docx]

Table S1: Primers used to construct Φ^2^DRMs

| Primer Name | Primer sequence | Fragment length |
| --- | --- | --- |
| pdnaK_ClaI | AGAGCTTATCGATGCTGCTCAGCGACCGTCAG | 307bp |
| pdnaK_SpeI | TTCTTACTAGTTCCTCCTGAATATGTAGAGCT |  |
| phsp_ClaI | AGAGCTTATCGATAGGGTATGAGGGGCAAATT | 207bp |
| phsp_SpeI | CTCCTTCTTACTAGTTCTCCTCATGCTTCGTT |  |
| phspX_ClaI | AGAGCTTATCGATCAACCTCCGCTGTTCGATA | 177bp |
| phspX_SpeI | TTCTTACTAGTAAACGGATGCCTTTGATCCGA |  |
| plat_ClaI | AGAGCTTATCGATGGCGAAGAGAGCTACGTCT | 201bp |
| plat_SpeI | TTCTTACTAGTCTATGATAGCAGGATTTACGG |  |
| pnarK2_ClaI | AGAGCTTATCGATGCTCCCCTTTCCAGTGGCG | 280bp |
| pnarK2_SpeI | TTCTTACTAGTCGATCCGGGGTCTCGGACTCC |  |
| pRv2034_ClaI | AGAGCTTATCGATAATACTAGGGAGCCTCTCT | 204bp |
| pRv2034_SpeI | TTCTTACTAGTAAGTTTAGACTTACGGATATT |  |
| pRv2623_ClaI | AGAGCTTATCGATACACATTCCGCAGGCATCG | 210bp |
| pRv2623_SpeI | TTCTTACTAGTGTCCTCCTGTCGTTGGCTGAT |  |
| pgroEL_ClaI | AGAGCTTATCGATGGATCCCGCTGGACGTTGC | 221bp |
| pgroEL_SpeI | TTCTTACTAGTCTACGCAGGGCTAGTCCGCTA |  |
|  |  |  |
